# Spontaneous regression of small cell lung cancer associated with Lambert-Eaton Myasthenic Syndrome: Case report

**DOI:** 10.1016/j.radcr.2023.08.059

**Published:** 2023-09-02

**Authors:** Michimi Otani, Miki Nishimori, Hitomi Iwasa, Mamiko Iwamura, Takayasu Izumi, Kosuke Nakaji, Noriko Nitta, Kana Miyatake, Rika Yoshimatsu, Tomoaki Yamanishi, Tomohiro Matsumoto, Yasushi Osaki, Noriko Wada, Makoto Toi, Marino Yamamoto, Yu Nakatani, Tetsuya Kubota, Takuji Yamagami

**Affiliations:** a Department of Diagnostic and Interventional Radiology, Kochi Medical School, Kochi University, Kohasu, Oko-cho, Nankoku, Kochi, 783-8505, Japan; b Department of Neurology, Kochi Medical School, Kochi University, Kohasu, Oko-cho, Nankoku, Kochi, Japan; c Department of Diagnostic Pathology, Kochi Medical School, Kochi University, Kohasu, Oko-cho, Nankoku, Kochi, Japan; d Department of Thoracic Surgery, Kochi Medical School, Kochi University, Kohasu, Oko-cho, Nankoku, Kochi, Japan; e Department of Respiratory Medicine and Allergology, Kochi Medical School, Kochi University, Kohasu, Oko-cho, Nankoku, Kochi, Japan

**Keywords:** Spontaneous regression, Small cell lung cancer, Lambert-Eaton myasthenic syndrome

## Abstract

Spontaneous regression (SR) of cancer is very rare, especially of small cell lung cancer (SCLC). Recently, an association of paraneoplastic neurological syndrome (PNS) has been reported as a cause of SR of cancer, and onconeural antibodies are a possible factor in the SR of cancer associated with PNS. We herein report the first case of SR of SCLC combined with anti-P/Q-type of voltage-gated calcium channel (VGCC) antibody-positive Lambert-Eaton myasthenic syndrome (LEMS), a subtype of PNS. This case report suggests that SCLC may be spontaneously reduced by an autoimmune response induced by VGCC antibodies associated with LEMS. Our finding may help elucidate the mechanisms that inhibit tumor growth and cause the regression of tumors.

## Introduction

Spontaneous regression (SR) of cancer is defined as the partial or complete disappearance of a malignant tumor in the absence of treatment or in the presence of therapy that is considered inadequate to produce a significant effect on the disease [1]. SR of cancer is very rare, especially in small cell lung cancer (SCLC) [2]. Its mechanism is poorly understood. However, an association between SR of cancer and paraneoplastic neurological syndrome (PNS) has been reported [2–5]. PNS is caused by tumor cells expressing neural system antigens which cross-immunize with nervous tissues. As a result, onconeural antibodies are produced, leading to neural system dysfunction. The onconeural antibodies are a possible cause of SR of tumors associated with PNS [3–7]. We herein report the first case of SR of SCLC combined with anti-P/Q-type of voltage-gated calcium channel (VGCC) antibody-positive Lambert-Eaton myasthenic syndrome (LEMS), a subtype of PNS, and discuss possible mechanisms that may explain this phenomenon.

## Case presentation

**Fig. 1:**
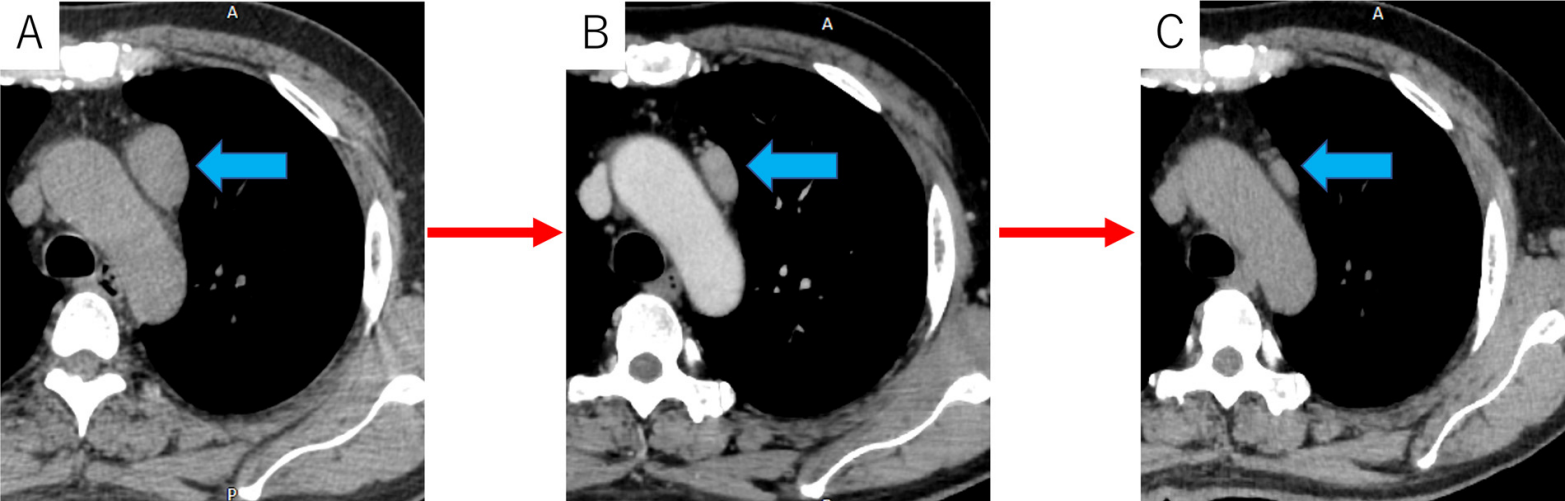
(A) The anterior mediastinum mass was smooth and 45 mm in maximum diameter on nonenhanced CT acquired at the hospital previous to the patient's consultation at our hospital (arrow). (B) One month later, contrast-enhanced CT of the chest during the patient's first visit to our hospital showed that the mass had regressed to 31 mm (arrow). (C) Another month later, a chest CT examination before surgery revealed further tumor regression to 27 mm (arrow).

A 56-year-old man was referred to our hospital for consultation regarding a 2-month history of whole-body weakness, double vision, difficulty swallowing, and an anterior mediastinal mass revealed by chest computed tomography (CT). The laboratory values on admission are shown in Table 1. The anterior mediastinum mass was 45 mm in maximum diameter and smooth as shown on nonenhanced CT obtained at the previous hospital. One month later, contrast-enhanced CT of the chest during the patient's first visit to our hospital showed that the mass had regressed to 31 mm. Enhancement was heterogeneous and the surrounding tissues were not involved. One month later, a chest CT performed before surgery revealed further tumor regression to 27 mm (Fig. 1). The patient had received no treatment during the 2-month interval since he had consulted with our hospital.

**Table 1:** Laboratory values on admission.

<Hematology>				<Biochemistry>				<Blood cogulation test>				<Serology>			
	Value	Reference	Unit		Value	Reference	Unit		Value	Reference	Unit		Value	Reference	Unit
WBC	10.2	3.3-8.6	10^3/μL	TP	6.8	6.6-8.1	g/dL	APTT	25.4	22.5-37.5	sec	CRP	0.05	≦0.14	mg/dL
BAND	1	0-19	%	Alb	4.5	4.1-5.1	g/dL	PT	10.4	9.8-12.1	sec	CEA	2.2	<5.0	ng/mL
SEGMENT	80	28-68	%	T-Bil	0.4	0.4-1.5	mg/dL	PT-INR	0.95	0.9-1.1		CYFRA	1.3	<3.5	ng/mL
LYMPHO	11	17-57	%	AST	21	13-30	U/L					SCC	0.5	<1.5	ng/mL
MONO	6	0-10	%	ALT	33	10-42	U/L								
EOSINO	2	0-10	%	LDH	226	124-222	U/L								
BASO	0	0-2	%	ALP	57	38-113	U/L								
RBC	493	435-555	10^4/μL	γ-GTP	48	13-64	U/L								
Hb	17	13.7-16.8	g/dL	BUN	12.9	8-20	mg/dL								
Ht	49.4	40.7-50.1	%	Cr	0.44	0.65-1.07	mg/dL								
PLT	25	15.8-34.8	10^4/μL	Na	143	138-145	mmol/L								
				K	4	3.6-4.8	mmol/L								
				Cl	105	101-108	mmol/L								
				Ca	9.6	8.8-10.1	mmol/L								
				HbA1c	6.7	4.9-6.0	%								

Alb, albumin; ALP, alkaline phosphatase; ALT, alanine aminotransferase; APTT, activated partial tromboplastin time; AST, aspartate aminotransferase; BUN, blood urea nitrogen; Ca, calcium; CEA, carcinoembryonic antigen; Cl, chloride; Cr, creatinine; CRP, C-reactive protein; CYFRA, cytokeratin fragment21; Hb, hemoglobin; HbA1c, hemoglobin; Ht, hematocrit; K, potassium; LDH, lactate dehydrogenase; Na, sodium; PLT, platelet count; PT, prothrombin time; PT-INR, prothrombin time-international normalized ratio; RBC, red blood cell count; SCC, squamous cell carcinoma-related antigen; T-bil, total bilirubin; TP, total protein; WBC, white blood cell count; γ-GTP, gamma-glutamyl transferase.

Neurological examination showed proximal muscle leg weakness and depression of tendon reflexes. Electromyography demonstrated the compound muscle action potential amplitude of the abductor digiti minimi muscle (2.0 mV) increased by 6-fold (12.1 mV) after 20 seconds of maximal contraction. Repetitive nerve stimulation at 1-10 Hz showed over 19% decrement in the abductor digiti minimi muscle. These results were consistent with a presynaptic neuromuscular transmission disorder. Laboratory testing revealed anti-P/Q-type VGCC antibodies (≧30 pmol/L). Therefore, we made a diagnosis of LEMS.

**Fig. 2:**
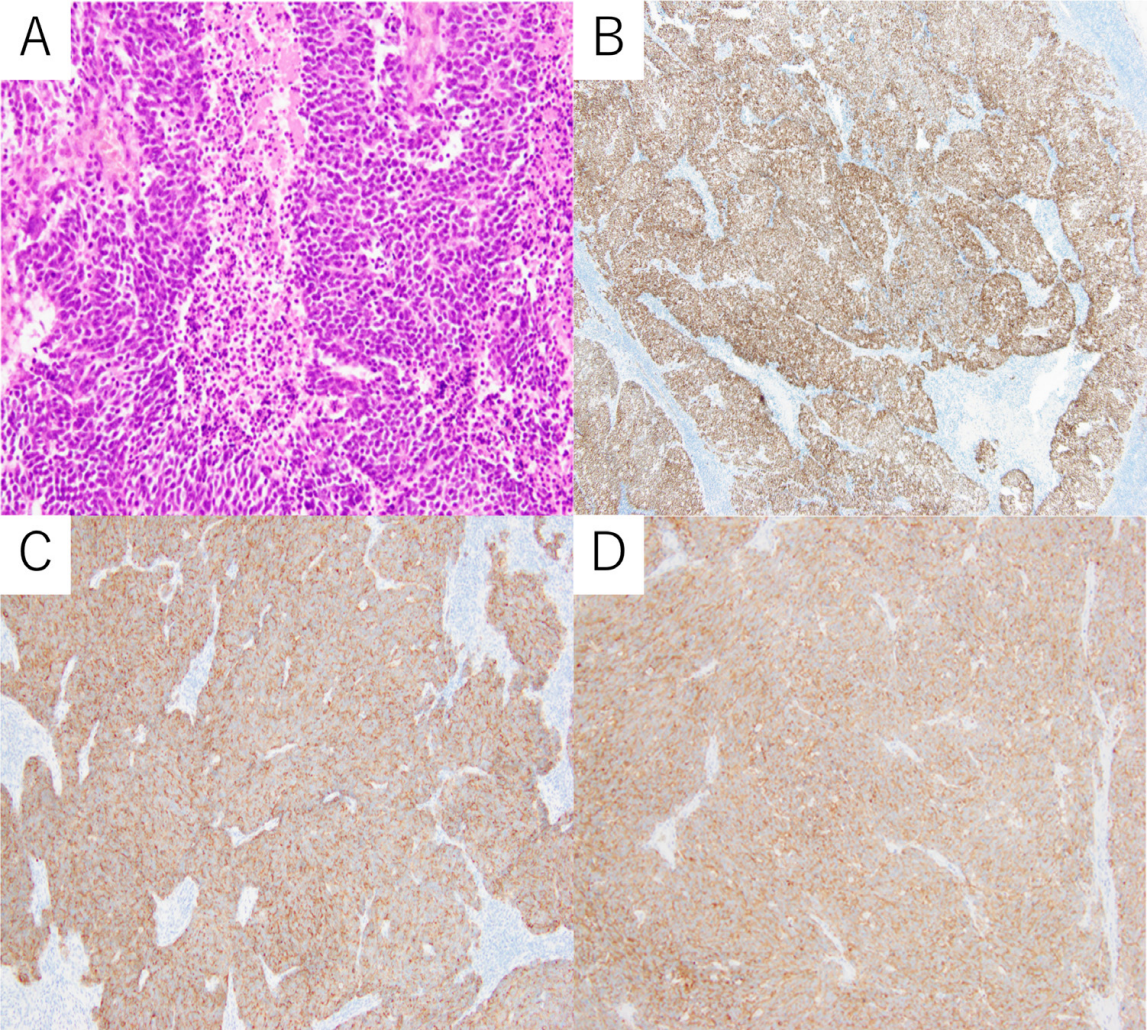
(A) Histopathological examination showed growth of small round to oval-shaped tumor cells with scant cytoplasm, poorly defined cell borders, finely granular nuclear chromatin, a high nuclear-to-cytoplasmic ratio, and a high mitotic index. The tumor cells grew in a sheet-like pattern with rosette-like structures and areas of necrosis. Immunohistochemical analysis revealed positive staining for (B) TTF-1, (C) chromogranin A, and (D) synaptophysin.

Under the diagnosis of an anterior mediastinal tumor associated with LEMS, tumorectomy was performed by video-assisted thoracic surgery. Histopathological examination showed growth of small round to oval-shaped tumor cells with scant cytoplasm, poorly defined cell borders, finely granular nuclear chromatin, a high nuclear-to-cytoplasmic ratio, and a high mitotic index. The tumor cells grew in a sheet-like pattern with rosette-like structures and areas of necrosis. Immunohistochemical analysis revealed positive staining for AE1/AE3, TTF-1, chromogranin A, and synaptophysin (Fig. 2). The most likely diagnosis based on these findings was lymph node metastases from small cell cancer.

**Fig. 3:**
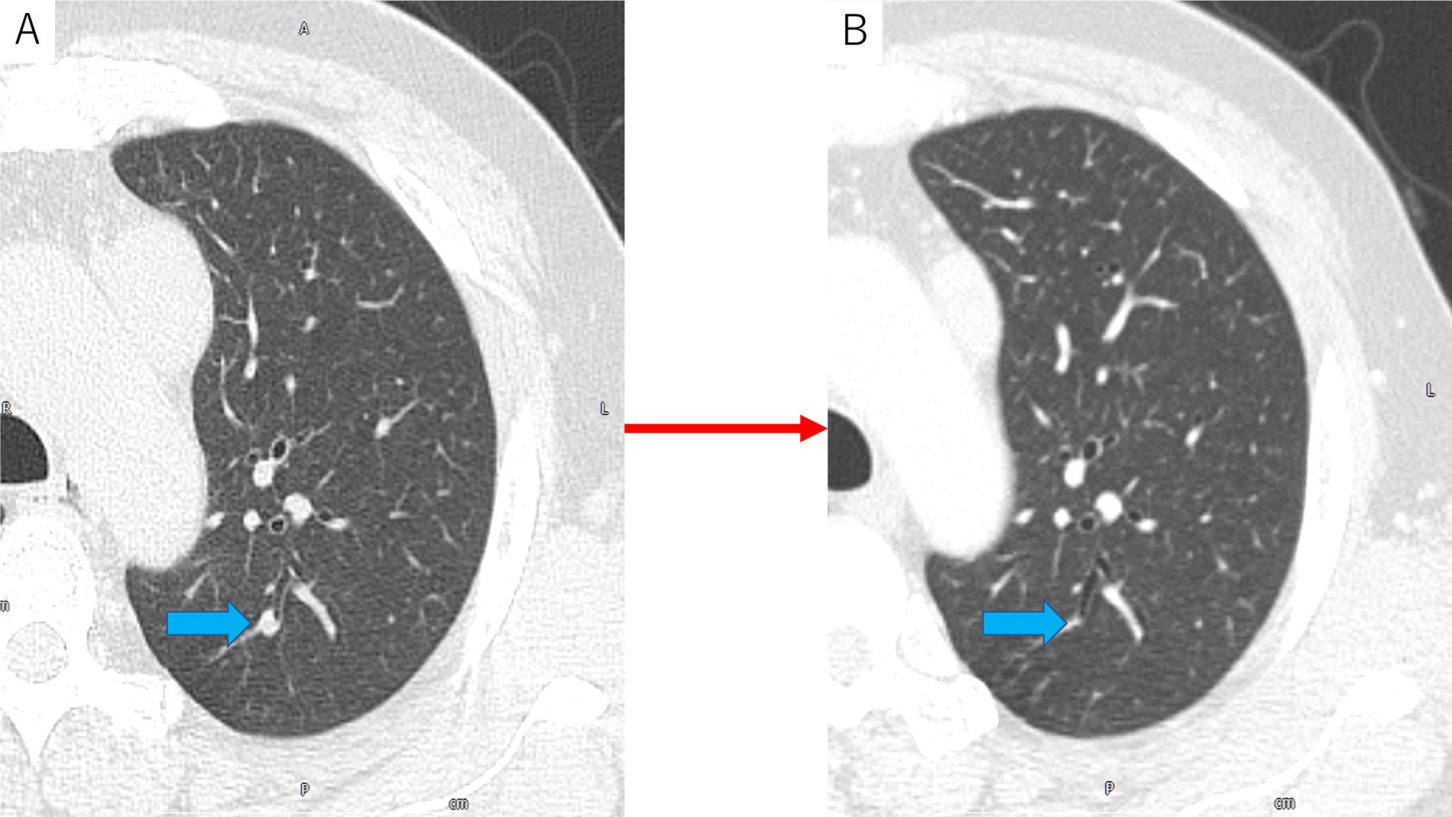
(A) CT examination at the patient's previous hospital revealed a pulmonary nodule of 4.5 mm in the left upper lobe (arrow). (B) Chest CT at the first presentation at our hospital revealed disappearance of the nodule (arrow).

After surgery, the previously obtained images were re-evaluated. The CT examination at the previous hospital revealed a pulmonary nodule of 4.5 mm in the left upper lobe. Chest CT at the first presentation at our hospital revealed disappearance of the nodule (Fig. 3). The patient was finally suspected of having SCLC with anterior mediastinal lymph node metastasis complicated by LEMS based on clinical, imaging, and pathology findings. The tumor markers neuron-specific enolase and pro-gastrin-releasing peptide were examined after diagnosis and were within normal range.

A ^18^F-fluorodeoxyglucose positron emission tomography-computed tomography (FDG-PET/CT) was performed after surgery. There were no primary lesions or metastases, even in the lungs. Neurological symptoms improved immediately following surgery. Additional platinum-based chemotherapy was performed. Immune checkpoint inhibitors were not used because of LEMS complications. There have been no signs of recurrence 2 year after surgery.

## Discussion

We herein report the first case of SR of SCLC combined with anti-P/Q-type VGCC antibody-positive LEMS. Although SR has been reported in association with PNS, there have been no reports of LEMS associated with SR of tumor [2–5]. Our findings suggest that autoimmune mechanisms associated with LEMS may be related to the SR of the tumor.

The SR of tumors is extremely rare, occurring in 1/60,000-100,000 cancer patients worldwide [8]. Although its mechanism is unclear, immune mechanisms, tumor necrosis, apoptosis, surgical trauma, biopsy, infection, hormonal issues and induction of benign differentiation have been considered [9–11]. SR is more commonly associated with kidney cancer, neuroblastoma, and malignant melanoma [12] with SR of lung cancer rarely reported. Reports of SR of SCLC are even rare [10]. In a review of case reports of SR of lung carcinoma from 1988 to 2018, Zhang et al. [12] found only 14 cases of spontaneously regressed lung cancer including SCLC and nonsmall cell lung cancer, with only 3 cases having SCLC.

There have been reports of SR of cancer associated with PNS [2–5]. Onconeural antibodies are a possible cause of SR of tumors associated with PNS [3–7]. Zhang et al. [12] found that among 14 cases who had spontaneously regressed lung carcinoma were patients with PNS, some who were positive for onconeural antibodies. PNS is among a group of neurological disorders [13] triggered by a distant tumor and not directly caused by cancer metastasis, side effects of cancer treatment, nutritional deficiencies, metabolic derangements, or coagulopathies [14]. PNS arises in the context of an immune response generated against antigens expressed on tumor cells that are also expressed in the nervous system [15]. An onconeural antibody is created that targets tumors to combat the growing malignancy [12]. These onconeural antibodies and associated onconeural antigen-specific T cells mistakenly assault parts of the nervous system because of antigenic similarities, inducing a variety of immunological responses that result in an immune-mediated neural syndrome [12]. The association of PNS and onconeural antibodies with SR of tumor suggests that those antitumor immune-mediated responses are a potential mechanism for tumor regression and that PNS may promote an antitumor immune response by affecting autoimmunity in the tumor [12,16,17]. In our case, the patient had LEMS, which is a PNS [18].

LEMS is an autoimmune disorder of the neuromuscular junction caused by antibodies produced against the P/Q-type VGCC in the presynaptic neuronal cell membrane, thus reducing the release of acetylcholine from presynaptic nerve terminals [19,20]. Clinically, LEMS is characterized by proximal muscle weakness, dysautonomia, and reduced or absent deep tendon reflexes [21]. About 60% of LEMS is manifested as a paraneoplastic disorder (SCLC-LEMS), most commonly in association with SCLC [22]. SCLC cells have been shown to express high concentrations of functional P/Q-type VGCC, which presumably induce autoimmune production of pathogenic anti-VGCC antibodies [22]. Approximately 90% of persons with LEMS present with P/Q VGCC antibodies [23], as did the current case.

To date, there have been reports of cancer-associated retinopathy [4], paraneoplastic sensory neuronopathy [2,5], paraneoplastic sensorimotor neuronopathy [6], and hemichorea [3] as PNSs associated with SR of a tumor but LEMS has not been reported. Also although there have been reports of anti-Hu antibodies [5], [6], [7], anti-Yo antibodies [7], anti-SOX1 antibodies and anti-CV2/CRMP5 antibodies [3], and anti-recoverin antibodies [4] as onconeural antibodies associated with PNS related SR, there have been no reports of VGCC antibodies. However, SCLC patients with clinical symptoms and signs of LEMS and positivity for VGCC antibodies had improved survival were reported [24,25]. Maddison et al. suggested that serum factors such as VGCC antibodies from patients with LEMS-SCLC are capable of reducing SCLC tumor cell proliferation, perhaps as a result of cell-surface binding to VGCCs and, specifically, that only pathogenically functional VGCC antibodies can act on channels on SCLC cells, reducing tumor proliferation [25]. The SR in our case may be related to these immunologic responses. Unfortunately, in the present case, we did not attempt to identify other onconeural antibodies, such as serum titers of SOX1 antibody [26], which are similarly associated with LEMS and associated with SR. Therefore, it is unclear whether VGCC antibodies alone were responsible for the SR of cancer in the present patient. However, we have shown for the first time that the autoimmune effects of VGCC antibodies associated with LEMS may be associated with SR of SCLC.

## Conclusion

We report for the first time a case of SR of SCLC combined with P/Q-type VGCC antibody-positive LEMS. This case report suggests that SCLC may be spontaneously reduced by an autoimmune response induced by VGCC antibodies associated with LEMS. This finding may help elucidate the mechanisms of tumor immunity and mechanisms that inhibit tumor growth and regress tumors. Such findings may play an important role in the development of immunotherapy against tumors. Because there have been no reports of SR of tumors associated with LEMS, it seems that the actual relationship among PNS, VGCC antibodies, and SR of lung cancer still needs to be clarified by further evidence with more cases.

## Ethics committee approval

This is a case report involving 1 patient; thus, institutional ethics committee approval was not required.

## Patient consent

Written informed consent was obtained from the patient described in this manuscript.
